# Enteral nutrition products in ICUs: data from NutritionDay

**DOI:** 10.1186/cc9800

**Published:** 2011-03-11

**Authors:** B Mora, M Mouhieddine, P Singer, S Ruiz-Santana, M Hiesmayr

**Affiliations:** 1 Medical University of Vienna, Austria; 2 Rabin Medical Center, Tel Aviv University, Tel Aviv, Israel; 3 Hospital Universitario de Gran Canaria Dr Negrín, Las Palmas de Gran Canaria, Spain

## Introduction

Pharmaceutical companies have introduced to the market many products for enteral nutrition. The different products offer a wide variety of compositions or have specific macronutrients or micronutrients added and are marketed for specific patient groups or conditions. Thus an individualised therapy may be associated with the use of a wide variety of products. For practical reasons, easier stock management, economic reasons, increased experience and error prevention, a standardised nutritional care would be more common practice. It is unknown to which extent these two options are applied in clinical practice. We have investigated the uses and behaviours about nutritional products in different ICUs from the data of NutritionDay (ND).

## Methods

The ICU ND is an ECCRN-supported cross-sectional audit in 10 languages. We have analysed from the 4-year database which enteral products were received by patients enrolled from 2007 to 2010 in the ND study. The aim of our study was to find which and how many different enteral products are given in each ICU.

## Results

**Figure 1:**
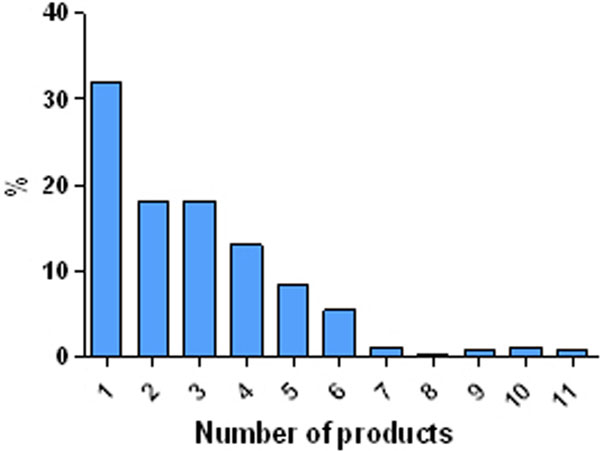
**Relationship products/ICU**.

**Figure 2:**
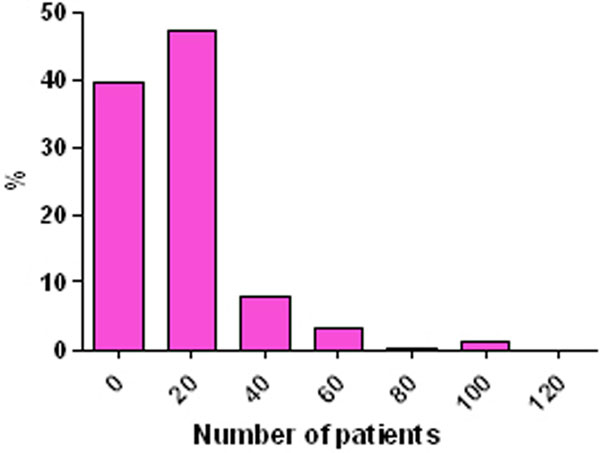
**Relationship patients/ICU**.

Two hundred different enteral products have been used in 237 ICUs. Nearly 50% of ICUs used one or two products (Figure 1). Most ICUs recruited 10 to 30 patients during the ICU ND audits (Figure 2).

## Conclusions

There is a very huge offer of enteral nutrition products but it is very common that in most ICUs (almost 70%) only one to three different products have been given. Individualisation of nutrition therapy in terms of diet composition is not common practice.

